# Assessment of neuromuscular and haemodynamic activity in individuals with and without chronic low back pain

**DOI:** 10.1186/1476-5918-5-6

**Published:** 2006-05-31

**Authors:** Melissa D McKeon, Wayne J Albert, J Patrick Neary

**Affiliations:** 1 Human Performance Laboratory, Faculty of Kinesiology, University of New Brunswick, Fredericton, Canada; 2 Faculty of Kinesiology and Health Studies, University of Regina, Saskatchewan, Canada

## Abstract

**Background:**

Biering-Sørenson (1984) found that individuals with less lumbar extensor muscle endurance had an increased occurrence of first episode low back pain. As a result, back endurance tests have been recommended for inclusion in health assessment protocols. However, different studies have reported markedly different values for endurance times, leading some researchers to believe that the back is receiving support from the biceps femoris and gluteus maximus. Therefore, this study was designed to examine the haemodynamic and neuromuscular activity of the erector spinae, biceps femoris, and gluteus maximus musculature during the Biering-Sørenson Muscular Endurance Test (BSME).

**Methods:**

Seventeen healthy individuals and 46 individuals with chronic low back pain performed the Biering-Sørenson Muscular Endurance Test while surface electromyography was used to quantify neuromuscular activity. Disposable silver-silver-chloride electrodes were placed in a bipolar arrangement over the right or left biceps femoris, gluteus maximus, and the lumbosacral paraspinal muscles at the level of L_3_. Near Infrared Spectroscopy was used simultaneously to measure tissue oxygenation and blood volume changes of the erector spinae and biceps femoris.

**Results:**

The healthy group displayed a significantly longer time to fatigue (Healthy: 168.5s, LBP: 111.1s; p ≤ 0.05). Significant differences were shown in the median frequency slope of the erector spinae between the two groups at 90–100% of the time to fatigue while no significant differences were noted in the haemodynamic data for the two groups.

**Conclusion:**

Although the BSME has been recognized as a test for back endurance, individuals with chronic LBP appear to incorporate a strategy that may help support the back musculature by utilizing the biceps femoris and gluteus maximus to a greater degree than their healthy counterparts.

## Background

The issue of low back pain in society is overwhelming. The Manga report conducted by the University of Ottawa, Canada (1993) stated that low back pain (LBP) is the leading cause of disability and morbidity in middle-aged persons and is by far the most expensive source of workers' compensation costs in North America [1]. The economic impact for Canada is staggering with direct and indirect costs associated with LBP incidence being quoted as high as 800 million and 3 billion dollars, respectively [1] This translates into approximately 35% of all compensation claims being related to LBP incidences [1].

In the majority of LBP cases, pain associated with an initial episode resolves within 2 to 4 weeks [2]. However, 2 to 3% of this population will develop chronic symptoms while the rest of the population may be susceptible to recurrent episodes of LBP. Within the first year after an acute episode, it has been documented that recurrence rates may range anywhere from 60 to 86% [3]. With recurrence rates being so high, it is paramount to determine suitable assessment procedures and treatments to restore proper muscle function and activities of daily living.

In 1984 Biering-Sørenson found that individuals with less lumbar extensor muscle endurance had an increased occurrence of first episode low back pain [4]. As a result, back endurance tests have been recommended for inclusion in health assessment protocols [5].

Several techniques for examining extensor muscle fatigue have been identified in the literature. In studies by Nicolaisen and Jørgenson [6,7], two specific tests were outlined; the first test measured the length of time a subject was able to sustain a 60% maximal voluntary contraction of the trunk muscles against a dynamometer in a vertical position. The second measured how long an individual was able to support their torso in a horizontal position. Though Nicolaisen and Jørgenson [7] found the first method to be the most effective in their research, many recent studies have examined the use of the second method, known as the Biering-Sørenson Muscular Endurance test (BSME). Recent literature has demonstrated the BSME to provide reliable measures with the ability to discriminate between individuals with and without LBP [8]. The BSME has also been shown to be unaffected by the level of physical activity that an individual engages in [8]; thus making the test appropriate for an entire population and therefore maintaining a high external validity.

Previous research has also used quantitative methods to examine LBP. For example, electromyographical (EMG) techniques have been used to document neuromuscular fatigue, defined as "a reduction in maximal force production of the muscle" and characterized by a "reduced ability to perform work" [9]. In particular, the erector spinae musculature has been examined extensively. Different studies have reported markedly different values for endurance times; particularly between males and females. It has been demonstrated previously that females have significantly greater erector spinae endurance than males [4,10,11]. This longer endurance time has been attributed to a greater presence of type I fibres in the lumbar region of females [12]. However, this may not be the sole reason for an extended endurance time; instead the back may be receiving support from the biceps femoris and gluteus maximus [5] which are key stabilizers of the trunk.

Several researchers have recently demonstrated differences in metabolic fatigue between healthy individuals and individuals with LBP [13,14] but not all studies support this contention [15]. Metabolic fatigue in these studies was examined using near infrared spectroscopy (NIRS) [16] a non-invasive optical technique based upon a modified *Beer-Lambert Law *[17]. Near infrared spectroscopy provides researchers with the ability to monitor tissue oxygenation which is defined as "the relative saturation of oxyhemoglobin and oxymyoglobin" and relies on the "balance between oxygen delivery and oxygen consumption" [18]. Using NIRS, Kovacs et al. [13] found significant differences in the usage of oxygen of the erector spinae muscles, and Kell [14] demonstrated that healthy subjects appeared to have a greater ability than LBP subjects to extract oxygen from the erector spinae muscles. However, Kankaanppa et al. [15] did not find significant differences in muscle oxygenation or MF slope between the healthy and LBP individuals during a dynamic lumbar (isoinertial) endurance test. The differences in back endurance test protocols may account for the variation in the results of the studies.

Therefore, the purpose of this study was to examine the myoelectrical activity and fatigue characteristics of the erector spinae, biceps femoris, and gluteus maximus musculature in both males and females performing the BSME Test. It was hypothesized that significant differences would be found between the healthy and LBP groups for all EMG and NIRS variables.

## Methods

### Participants

Seventeen healthy subjects (8 males and 9 females) 46 subjects reporting chronic low back pain (LBP) (17 males and 29 females) volunteered to perform the BSME test for assessment of neuromuscular activity (EMG). The healthy population had a mean age, height, weight and BMI of 24.6 ± 5.0 years, 1.73 ± 0.07 m, 73.18 ± 15.26 kg, 24.30 ± 3.76 kg/m^2^, respectively. The chronic low back group had mean age, height, weight and BMI of 42.7 ± 13.7 years, 1.73 ± 0.11 m, 81.4 ± 14.69 kg, 27.25 ± 4.49 kg/m^2^, respectively. Sixteen of these healthy subjects (8 males and 8 females) and 27 of the subjects reporting chronic LBP (10 males and 17 females) volunteered for the assessment of haemodynamic activity during the BSME. All individuals were required to read and sign an informed consent form approved by the institution's ethics review board.

### Questionniares

All subjects reporting low back pain were required to complete the Oswestry Disability Index and the Roland Morris Disability Questionnaire to assess their degree of LBP [19]; while all healthy subjects were interviewed and verbally screened for prior history of LBP.

### Biering-Sørenson muscular endurance test

The Biering-Sørenson muscle endurance test (BSME) test was performed in accordance with the Canadian Physical Fitness and Lifestyle Appraisal guidelines [20]. Subjects were asked to lye in a prone position on a table. The subject's upper body was positioned with their iliac crests at the edge of the table; with lower body secured at the ankles and hamstring level using seatbelt straps. Arms were held across the chest with hands placed on the opposite shoulder as a horizontal position was held until exhaustion was reached. The test was discontinued once the subjects could no longer maintain a horizontal position level to the table. One chance was given for the subjects to reposition their upper body during the test while standard verbalized encouragement was given throughout for all subjects.

### Electromyography (EMG)

To monitor electromyographic activity during the BSME test, disposable silver-silver-chloride Red Dot™ electrodes were placed in a bipolar arrangement over the right or left biceps femoris, one-third to midway along a line connecting the fibular head with the ischial tuberosity; gluteus maximus, approximately 10 cm inferior to the posterior superior iliac spine; and the lumbosacral paraspinal muscles, approximately 2–3 cm lateral to the spinous processes at a level of L_3_. The right or left side placement of the electrodes was randomly chosen for each healthy individual and was kept consistent for all muscle groups while right side placement was consistently chosen for the LBP subjects due to the variable location of each individual's pain. This protocol is supported by recent studies showing no significant differences between right and left side electromyographic activity in subjects with and without LBP [15].

The signal was gathered at a sample rate of 2000 Hz, amplified with a gain of 1000, and was A/D converted with data acquisition software (DI-720, DATAQ Instruments Inc, Akron, Ohio, USA). Analysis of the EMG signal was performed using a custom built Labview 6.1 program (National Instruments, Texas, USA). The raw EMG data was processed using a zero lag 4^th ^order band pass Butterworth filter with low and high cut-off frequencies of 20 Hz and 500 Hz. To determine the average median frequency (MF) of each fatiguing contraction, the signal was broken into overlapping epochs of 0.25 second and averaged for each one second throughout the endurance test. All data was normalized to a start value of zero and averaged across all participants. MF slope values were obtained using a line of best fit and a one-way analysis of variance (ANOVA) was then used to calculate differences in MF slopes.

### Near Infrared Spectroscopy (NIRS)

Throughout the BSME, a Spatial Resolved (SRS) Near Infrared Spectrometer (NIRO-300, Hamamatsu Photonics K.K, Japan) was used to examine muscle oxygenation and blood volume changes of the erector spinae and biceps femoris muscles. The NIRO-300 instrumentation uses a laser at four different wavelengths (775, 810, 850, 905 nm) to calculate the relative quantitative concentration changes from baseline in micromolar (mM) units. The sum of the oxygenated and deoxygentated haemoglobin signal indicates the change in total blood volume (combined haemoglobin: cHb), while total oxygen index (TOI) is a measure of oxygen saturation (TOI = HbO_2_/cHb) [18]. A modified Beer-Lambert Law was used in the calculation of these parameters [17].

The NIRS photo-detectors were placed over the biceps femoris, one-third to midway along a line connecting the fibular head with the ischial tuberosity, and on the lumbosacral paraspinal muscles approximately 2–3 cm lateral to the spinous processes at the level of L_3_. The interoptode spacing between the sensor and detector probe was 5 cm, providing a penetration depth of approximately 2.5 cm [21]. The optodes were fixed in place and secured to the skin (double adhesive tape) by using the manufacturer's custom-designed optically dense black holder, which also shield any extraneous room light. The NIRS photo-detectors were placed on the opposite side to the electromyography electrodes for all subjects. Prior to performing the BSME a two-minute baseline was collected where the subject lay prone with their arms resting by their sides. The changes in cHb and TOI were then collected and examined every 1 second throughout the static endurance test, and for a 100 second recovery phase. All data was normalized to 10% intervals for the duration of the test for standardization purposes, and for comparison between individuals [22].

### Data analysis

All raw data was analysed using a custom built Labview 6.1 program (National Instruments, Texas, USA). Mean and standard deviations were used to describe all variables. A series of one-way analyses of variance (ANOVA) were used to determine main and interaction effects while Tukeys' post hoc or Dunnett's C comparisons were used to detect significant differences when ANOVA effects were significant (*p *≤ 0.05). All ANOVAs were conducted independently of one another to reduce the possibility of a Type I error.

## Results

### Biering-Sørenson muscular endurance time

The independent variable, sex, included two levels: male and female, while the dependent variable was the time taken to complete the BSME test. Significant differences were noted between the healthy vs. LBP groups (*p *= 0.002), healthy males vs. females (*p *= 0.01), and healthy vs. LBP females (*p *= 0.001). Table 1 outlines the results of this analysis and the reported significance level.

**Table 1:** Group and gender comparisons of BSME times (seconds).

**VARIABLE**	**MEAN**	**DF**	**F**	**P**	**η2**
**Healthy: Group**	**168.5**	**(1, 59)**	**10.263**	0.002*	**0.148**
**Low Back Pain (LBP) : Group**	**111.1**				
					
**Healthy: Males**	**124.4**	**(1, 15)**	**8.22**	0.01*	**0.35**
**Healthy: Females**	**212.6**				
					
LBP: Males	115.3	(1, 42)	0.198	0.66	0.005
LBP: Females	106.5				
					
Healthy: Males	124.4	(1, 23)	0.0557	0.463	0.02
LBP: Males	115.3				
					
**Healthy: Females**	**212.6**	**(1, 36)**	**13.068**	0.001*	**0.26**
**LBP: Females**	**106.5**				

* Significance at *p *≤ 0.05

### Electromyography (EMG)

#### Gender (Sex) comparison

Significant differences in MF slope declines were found for the erector spinae and biceps femoris muscles for healthy males while significant differences in declines were found between the erector spinae and gluteus maximus muscles for healthy females. Significance differences in MF slope declines were found for the erector spinae and gluteus maximus muscles for LBP males while significant differences were found between the biceps femoris and gluteus maximus muscles for LBP females. Tables 2 and 3 display the muscle MF comparisons and their significance.

**Table 2:** Between muscle comparisons of the median frequency slopes during the BSME test in healthy individuals. Associated P-values for male and female subjects are reported.

	**Erector Spinae (ES)**		**Gluteus Maximus (GM)**		**Biceps Femoris (BF)**	
	**Male**	**Female**	**Male**	**Female**	**Male**	**Female**
**ES**	~~	~~	0.381	0.004*	0.005*	0.176
**GM**	0.381	0.004*	~~	~~	0.174	0.28
**BF**	0.005*	0.176	0.174	0.28	~~	~~

* Significance at *p *≤ 0.05

**Table 3:** Between muscle comparisons of the median frequency slopes during the BSME test in chronic low back pain individuals. Associated P-values for male and female subjects are reported.

	**Erector Spinae (ES)**		**Gluteus Maximus (GM)**		**Biceps Femoris (BF)**	
	**Male**	**Female**	**Male**	**Female**	**Male**	**Female**
**ES**	~~	~~	0.004*	0.202	0.07	0.289
**GM**	0.004*	0.202	~~	~~	0.825	0.002*
**BF**	0.07	0.289	0.825	0.002*	~~	~~

* Significance at *p *≤ 0.05

#### Healthy vs. LBP group and gender comparisons

**Figure 1:**
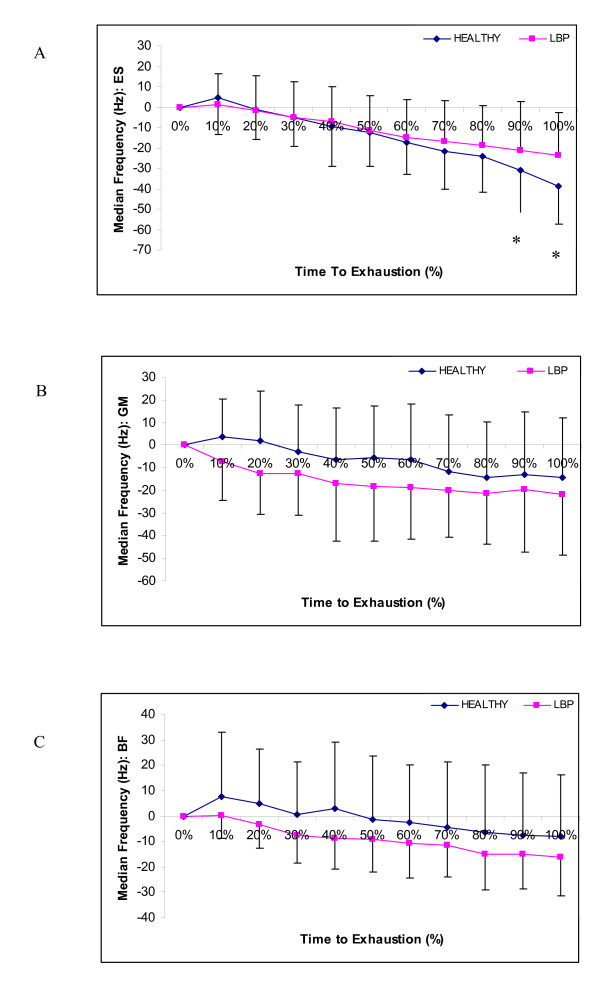
**Comparison of healthy and LBP Median Frequency slopes**. (A) Erector Spinae, (B) Gluteus Maximus, and (C) Biceps Femoris. In all graphs, time to exhaustion was normalized to 100% for direct comparison between subjects and groups. Error bars indicate one standard deviation of the mean. Using a one-way ANOVA, significant differences in MF were found in the erector spinae muscle at 90–100% of the performance time while no significant differences were found in either the gluteus maximus or biceps femoris muscles.

Figure 1 displays the comparison of healthy and LBP MF for each of the 3 muscle groups. In all graphs, time to exhaustion was normalized to 100% for direct comparison between subjects and groups. Error bars indicate one standard deviation of the mean. Using a one-way ANOVA, significant differences in MF were found in the erector spinae muscle at 90–100% of the performance time while no significant differences were found in either the gluteus maximus or biceps femoris muscles.

Significant differences were found in the MF slopes of the healthy and LBP erector spinae muscles. Significant differences were also found in the gluteus maximus muscle of healthy and LBP females. Table 4 displays the muscle MF comparisons and their statistical significance.

**Table 4:** Comparison of median frequency slope values between the healthy and chronic low back pain groups for each muscle during the BSME test.

		**HEALTHY**		
		**Males**	**Females**	**Group Total**
**LBP**	**Erector Spinae**	0.059^a^	0.074	0.008*
	**Gluteus Maximus**	0.093	0.005*	0.321
	**Biceps Femoris**	0.125	0.12	0.942

*Significance at *p *≤ 0.05

^a ^Approaching significance

### Haemodynamics

**Figure 2:**
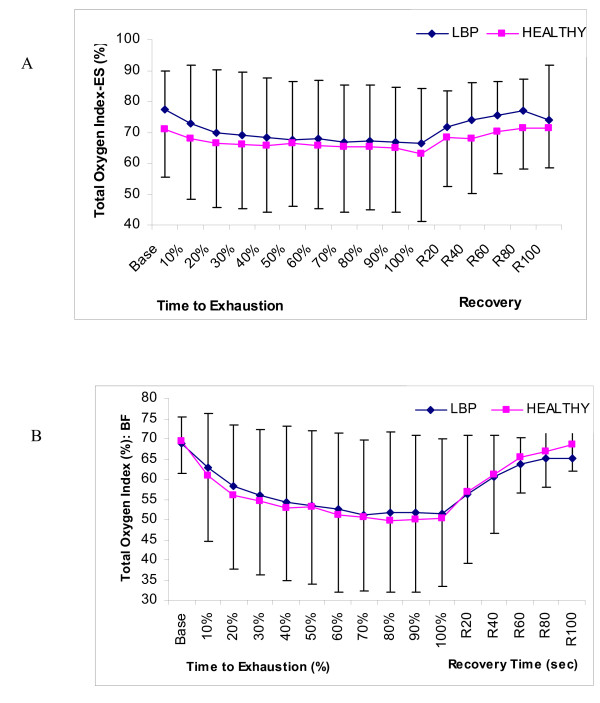
**Comparison of Total Oxygen Index in healthy and LBP subjects**. (A) Erector Spinae, and (B) Biceps Femoris.

No significant differences were found between the Healthy and LBP groups for either of dependent variables, TOI or the localized cHb. Figure 2 displays the changes in TOI and cHb during the BSME test protocol.

## Discussion

### Biering-Sørenson muscular endurance time

As previously reported in the literature [5,6,11]. this study demonstrated that significant differences were evident between healthy male and female back extensor endurance times. The healthy male endurance times are within the normal range of previously reported studies [7,24,25], although slightly lower than reported by some [4,6,23,23]. However, healthy female endurance times (117.2–219 s) appear to be consistent with previous findings in the literature [4-6]. Both LBP male and female endurance times appear within the ranges reported by others [24]. Table 5 summarizes the BSME times from previous studies, and further indicates that a number of research groups have employed this testing protocol for both LBP and healthy subjects. Thus, confirming the validity and reliability of using the BSME test.

**Table 5:** Comparison of studies reporting Mean Endurance Times (seconds) for Biering-Sørenson Muscular Endurance Test in healthy individuals and low back pain populations.

	**Healthy**			**Low Back Pain**		
	**Males**	**Females**	**Combined**	**Males**	**Females**	**Combined**
Biering-Sørenson (1984)	198	197		163	177	
Nicolaisen & Jørgensen (1985)	184 [59]	219 [33]		148 [61]	146 [62]	
Jørgensen & Nicolaisen (1987)	184 (70–240)			148 (45–240)		
McQuade et al. (1988)						35.1
Holmström et al. (1992)	171.5 (119–266)			137.5 (21–253)		
Alantra et al. (1994)				98	89	
Moreland et al. (1997)	101 (54–56)					
McGill et al. (1999)	146 [51]	189 [60]	171 [60]			
Payne et al. (2000)	112.8 {4.1}	117.2 {4.8}		77.5 {9.5}	92.5	
McKeon et al (2006)	124	212		115	106	

Note: ( ) Reported ranges, [ ] Standard Deviations, { } Standard Error Adapted from [24]

In 1984 Biering-Sørenson [4] found that individuals with less lumbar extensor muscle endurance had an increased occurrence of first episode LBP. As well, Latimer and colleagues [8] have found individuals with LBP to have a lower static endurance time and found it to be a reliable measure in discriminating between subjects with and without LBP. The findings from the current study support these findings for females only. Our findings concur with Nicölaison and Jørgenson [7], who also failed to report a difference in back endurance between healthy and LBP males.

### Electromyography: median frequency

In the past, researchers have attributed longer endurance times in females to a greater presence of type I muscle fibres in the lumbar region [26]. However, our results suggest this may not be the sole reason for an extended endurance time and that the back is receiving support from the biceps femoris and gluteus maximus muscles. In both healthy and LBP groups, the MF was found to decline in all three muscle groups throughout the BSME. This suggests that all muscle groups are active throughout the endurance test. As expected, the greatest amount of fatigue was noted in the erector spinae muscles. Differences between the erector spinae and biceps femoris was found for healthy males while healthy females displayed significant differences in the median MF slopes of the erector spinae and gluteus maximus muscles. Muscle differences were also noted between the healthy and LBP group with LBP males demonstrating a significant difference between the erector spinae and gluteus maximus and females displaying a significant difference between the biceps femoris and gluteus maximus muscles.

It was found that the LBP group had a shallower erector spinae slope (23% drop) than that of the healthy group (42% drop) with statistical significance being shown at 90 and 100% of time to exhaustion. When correlated with the BSME time, it would be expected that the LBP group would have a steeper slope due to a more rapid fatigue rate, particularly in females as they demonstrate the greatest decline in BSME endurance times. While it would be expected that the healthy group would display a shallower decline as the rate of fatigue is slower. A possible explanation for this shallower decline in the LBP group may be due to the activity of the other muscle groups throughout the BSME. Latimer et al. [8] suggested that subjects with current LBP were more likely to stop the test due to fatigue, while asymptomatic subjects would terminate their test due to significant pain. This did not appear to be the case in the present study as both the healthy and LBP groups displayed a significant decline in median frequency, indicating that both groups significantly fatigued the erector spinae muscle during the BSME test.

Differences in the gluteus maximus muscle were noted between females of the two groups with LBP females displaying a greater MF slope than their healthy counterparts, providing evidence that LBP females used this muscle more than healthy females. This trend was also noted in LBP males, but significant differences were not found and likely due to the fact that three healthy males also displayed this trend; i.e., greater recruitment of the GM. With a larger sample size it may be possible to determine whether this is a common trend or if this is a predictor of future LBP. Thus, further research is needed to determine whether the muscle recruitment pattern is a determinant of LBP development or whether it is the LBP that dictates the muscle recruitment pattern.

### Near Infrared Spectroscopy: total oxygen index

The average TOI response for the erector spinae muscle demonstrated a continued utilization of oxygen (as reflected by the decline in oxygen saturation; from 70% to 62%) throughout the test in order to provide sufficient energy to perform the BSME protocol. These findings are consistent with the findings of Albert et al. [5] and Yoshitake et al. [27] who demonstrated an almost identical response (trend) in the erector spinae muscles of their subjects. The average TOI response for the biceps femoris muscle displayed a greater decline in muscle oxygenation (69% to 49%) until the end of the endurance test at which time it returned to baseline over the course of the recovery phase. This decrease throughout the BSME may be explained by 1) an increased cellular oxygen uptake in the mitochondria due to an increased metabolism of the working motor units, or 2) an increased intramuscular pressure, which would subsequently reduce blood and oxygen supply to the active motor units and is said to be one of the most important factors in low back muscle fatigue [27-29].

### Near Infrared Spectroscopy: combined haemoglobin (cHb)

For both the healthy and LBP groups, the average cHb response for the erector spinae muscle increased rapidly until approximately 20% then displayed a relatively steady plateau with only minor increases until exhaustion was reached. Throughout recovery cHb returned to baseline values. This is consistent with the findings of Albert et al. [5] whose study also demonstrated an initial increase in blood volume followed by a plateau until the end of the static endurance test in which the signal then returned towards baseline. These findings and the findings from the present study differed from that of Yoshitake et al. [27] who reported a decrease in blood volume throughout the fatiguing contraction. The average cHb for the biceps femoris muscle differed from the erector spinae muscle as it increased steadily until the end of the static endurance test in which it decreased steadily until the end of the recovery period.

Several researchers have recently demonstrated that near infrared spectroscopy can distinguish between healthy individuals and individuals with LBP [13,14]. Kovacs et al [13], examined oxygen use of the erector spinae muscle in healthy and LBP populations while simultaneously monitoring motion characteristics. Significant differences were found in the usage of oxygen, though no significant differences were noted in blood volume. In a study conducted by Kell [14], healthy subjects appeared to have a greater ability than LBP subjects to extract oxygen from the erector spinae muscles. As well, it was thought that if the erector spinae muscles of the LBP subjects were weaker, then these individuals must exert a greater percent of maximal force to support their upper body mass which may have lead to an earlier occlusion of the blood to the erector spinae muscle. However, no significant differences were found between the healthy and LBP group for either of the TOI or the cHb variables in our present study. A recent study by Kankaanpaa et al. [15] examined the paraspinal oxygen turnover and endurance capabilities of the erector spinae in the healthy and LBP males using a submaximal isoinertial back endurance test. This study also demonstrated no significant differences between healthy and LBP subject groups. However, their study did demonstrate that subcutaneous tissue thickness strongly influences the NIRS variables and may help to explain the lack of significant results in our present study.

## Conclusion

Although the BSME has been recognized as a valid test for back endurance, individuals with chronic LBP appear to incorporate a strategy that may help support the back musculature by utilizing the biceps femoris and gluteus maximus to a greater degree than their healthy counterparts. Despite differences in muscle recruitment, this did not result in differences to localized blood volume (cHb) or oxygen saturation (TOI) throughout the BSME test in the two subject groups. At this time, further research is needed to determine the utility of using NIRS as an assessment tool for chronic low back pain due to its high individual variability by assessing individual responses. Further research is also needed to conclude whether an individual's motor pattern determines their risk of LBP or if the LBP alters one's movement pattern.

## Authors' contributions

All authors contributed equally to this research study. MM was a graduate student that collected and analyzed the physiological data. WA wrote the research grant (NSERC) that supported this study and guided the design, data interpretation and analysis, as well provided editorial comments on all revisions. PN Assisted in the research design, analysis and interpretation of the near infrared spectroscopy data, and edited and approved the final manuscript.

**Figure 3:**
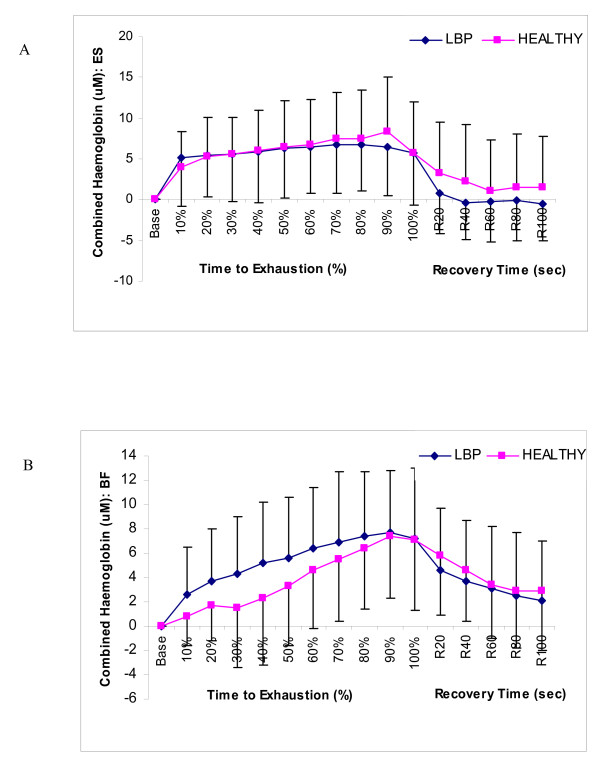
**Comparison of Combined Hemoglobin in healthy and LBP subjects**. (A) Erector Spinae, and (B) Biceps Femoris.
